# Movement Models to Predict Low‐Altitude Flight of Soaring Birds Using Look‐Ahead Environmental Factors

**DOI:** 10.1002/ece3.73059

**Published:** 2026-03-11

**Authors:** Rimple Sandhu, Charles Tripp, Eliot Quon, Regis Thedin, Michael Lanzone, Melissa A. Braham, Tricia A. Miller, Christopher J. Farmer, David Brandes, Todd Katzner

**Affiliations:** ^1^ National Laboratory of the Rockies Golden Colorado USA; ^2^ Cellular Tracking Technologies Rio Grande New Jersey USA; ^3^ Conservation Science Global Cape May New Jersey USA; ^4^ Orsted, Americas Newark New Jersey USA; ^5^ Lafayette College Easton Pennsylvania USA; ^6^ U.S. Geological Survey Boise Idaho USA; ^7^ School of Natural Resources and the Environment, West Virginia University Morgantown West Virginia USA

**Keywords:** data‐driven movement model, golden eagles, raptor conservation, telemetry data, turbine curtailment, wind–wildlife interactions

## Abstract

Advances in fine‐scale movement modeling of soaring birds can aid efforts to understand and resolve the impacts of anthropogenic activities on such birds. Soaring birds often rely on underlying terrain and low‐altitude updrafts to govern their flights at rotor‐swept altitudes (≤ 200 m above ground level), which puts them at risk of collision with wind turbines. We developed a data‐driven Markov model at 1‐s resolution that predicts the fine‐scale flight behavior of golden eagles ( *Aquila chrysaetos* ) as a function of ecological covariates at the current location as well as those within an eagle's line of sight. We only considered ecological covariates that are readily available in real‐time (ground elevation and wind conditions). Latent factors (age, sex, species, behavioral intent, migratory status) were intentionally left out of the model. We calibrated the model using golden eagle telemetry data collected in two different ecoregions of the United States. Given a starting location, the calibrated model simulates multiple stochastic 3D paths to produce a time‐explicit and spatially explicit risk map of turbine collisions. We discovered an empirical relation between the rate of change of heading and the orographic updraft conditions within an eagle's line of sight. Our model performed most effectively when predicting predominantly‐soaring flights at rotor‐swept altitudes during wind conditions in which turbines are likely to be operational. The calibrated model could be used in concert with automated eagle detection and turbine curtailment technologies. Specifically, once an eagle is detected by those systems, our model could then provide accurate predictions of turbines the eagle is likely to interact with in the near term.

## Graphical abstract

Eagles see ahead. Now our model does too. By modeling how eagles respond to terrain and wind conditions in their line of sight, we can predict where they'll fly in the next 3 min with 3× better accuracy than current methods—protecting eagles while maximizing energy production.

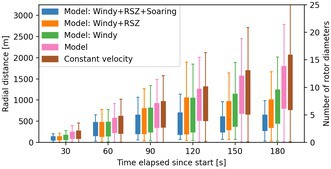

## 1 Introduction

Soaring raptors often rely on terrain‐induced updrafts to subsidize their low‐altitude flight, thereby putting them at a higher risk of collision with rotating wind turbines (hereafter “turbine collision”) (Miller et al. 2014; Marques et al. 2014; Katzner et al. 2019; Smallwood et al. 2008). Species of concern include golden eagle ( *Aquila chrysaetos* ; USA) (Smallwood et al. 2008; Katzner, Smith, et al. 2012; Katzner, Brandes, et al. 2012), bald eagle ( *Haliaeetus leucocephalus* ; USA) (Pagel 2013), griffon vultures ( *Gyps fulvus* ; Spain) (de Lucas et al. 2012), white‐tailed eagles ( *Haliaeetus albicilla* ; Germany, Norway) (Dahl et al. 2013; Barrios and Rodríguez 2004), and bearded vulture ( *Gypaetus barbatus* ; South Africa) (Reid et al. 2015). These species are often protected by law, further highlighting the importance of mitigating the risk of turbine collisions for these vulnerable and protected avian species. For example, eagles within the United States are protected under the Migratory Bird Treaty Act and the Bald and Golden Eagle Protection Act (United States 1940, 1918).

Assessments are often undertaken during the planning and siting stage of wind‐plant development to avoid potential regional‐level conflicts between raptor flight habitats and potential plant locations. However, raptor habitats often do not have well‐defined boundaries, cover a wide area, and often overlap with the land‐based wind resource potential (Miller et al. 2014; Katzner, Smith, et al. 2012; Katzner, Brandes, et al. 2012). This unavoidable situation makes it difficult to completely avoid overlap between raptor flight habitats and planned wind plants. Recently, research and effort have been focused on informed turbine curtailment strategies wherein an automated sensor‐based technology is used to detect a raptor flying toward a wind plant and specific turbines are stopped or slowed automatically (i.e., curtailed) to avoid a collision (McClure et al. 2018, 2021; Duerr et al. 2023). Potential turbines in conflict are determined using a predicted path of the detected eagle based on its initial location and flight speed, but not the topographical and environmental conditions affecting the predicted path of the eagle. Detection‐to‐curtailment response times are typically 10–30 s, meaning predictions must be made quickly once an eagle is detected. These operational constraints such as detection ranges of hundreds of meters, prediction horizons of 1–3 min, and turbine‐specific risk assessment are important considerations while building a real‐time predictive movement model of soaring raptors. However, this could lead to unnecessary curtailment, leading to lost energy production, or lack of curtailment, leading to fatalities of eagles. Recent work has highlighted important spatial variability in collision risk (therefore the frequency of curtailment) among turbines within a facility, likely from topographical and environmental conditions that affect the flight path of eagles (Duerr et al. 2023). To better protect eagles and maximize energy production, there is an interest to understand, model, and predict fine‐scale low‐altitude movements of soaring raptors in order to accurately predict and update the real‐time risk of turbine collision.

There exist at least two types of predictive modeling approaches to quantify the effect of ecological covariates on the movement dynamics of raptors. One approach involves data‐driven models such as correlated random walks (CRW) (Morales et al. 2004) and correlated velocity models (CVM) (Gurarie et al. 2017) that can incorporate different covariates at current or previous times/locations (Eisaguirre et al. 2019; Duerr et al. 2015). These approaches often involve explicit categorization of movement into a predefined set of flight modes (soaring/gliding/perching) and may incorporate eagle‐specific features (age, sex, species, behavioral intent, migratory status) into the model. For real‐time path prediction, limited information is often available about these latent features of the detected eagle. Also, when predicting or modeling fine‐scale movements over shorter time scales, discrete flight mode classification becomes practically challenging as eagles may transition rapidly between modes. Even if possible, such categorization may not be useful for short‐term movement prediction. Moreover, to our knowledge, these models have not been applied to incorporate directionally‐explicit look‐ahead covariates quantifying environmental conditions within an eagle's line of sight that may influence directional decisions before the eagle reaches those locations. Raptors such as golden eagles respond to the updrafts (or lack thereof) and terrain features in their line of sight with the aim of minimizing their energy expenditure, especially at altitudes that coincide with the rotor‐swept zone of wind turbines (Duerr et al. 2015; Katzner et al. 2015). For instance, while in flight powered by orographic updrafts (terrain‐induced upward moving air), a golden eagle might move towards a ridgeline hundreds of meters away and completely disregard the terrain and updraft conditions in the immediate vicinity. Therefore, models considering only the current or previous conditions may not be sufficient to build a robust predictive model of short‐term raptor behavior.

A second modeling approach uses agent‐based models that assume the simulated agent (the modeled bird) is fully aware of the conditions within an entire domain of interest that can encompass several square kilometers (Brandes and Ombalski 2004; Bohrer et al. 2012). This approach previously has mainly been applied to simulate the most likely energy‐minimizing routes for long‐term, directionally‐oriented soaring behavior including migration, which means the general direction of movement is known ahead of time. The recently published Stochastic Soaring Raptor Simulator (SSRS) used such an approach to simulate the likely horizontal paths of golden eagles traversing an orographic updraft field for a wind site in the western United States (Sandhu et al. 2022, 2021). However, when using real‐time avian detection technologies, the latent features of a detected raptor are not known, and the general direction of movement may be unknown or undefined. Also, the assumption that the simulated agent is completely aware of the updraft conditions kilometers away from its current location may be an oversimplification.

In this paper, our objective was to predict fine‐scale three‐dimensional (3D) movements of golden eagles using only the readily available environmental conditions (ground elevation and wind conditions). To accomplish this, we developed a modeling approach where movement dynamics are explicitly modeled as a function of conditions at both the current location and within a structured look‐ahead region representing the eagle's line of sight. While CRW and CRV models can theoretically incorporate covariates from non‐current locations, existing applications of these models for raptor movement have typically relied solely on local conditions or time‐lagged movement covariates. We implemented a discrete‐time Markov model as a function of ecological factors. In our model, parameters were estimated using 1‐s interval movement data from eastern and western United States. We particularly focused on movements with altitude above ground level (AGL) of less than 200 m, which is where most of the contemporary conflict between raptors and wind turbines occurs. We hereby refer to this region as the *rotor‐swept zone* (RSZ).

We used the proposed model to ask the following questions: (1) Is there a direct relation between the rate of change of heading of an eagle and the updraft conditions at locations along the line of sight? (2) Can the model calibrated using telemetry data from two topographically and climatically diverse regions of North America generalize well on any randomly selected telemetry track from those areas? (3) Can the calibrated model predict RSZ movements for up to 3 min from the time an eagle is initially detected by a curtailment system?

## 2 Materials and Methods

### 2.1 Study Species

We considered the golden eagles within the United States as our target raptor species for this study. Golden eagles are large birds that look for opportunities to exploit atmospheric updrafts to sustain their soaring flight (Duerr et al. 2019), making them an ideal study species. Our modeling effort used golden eagle telemetry data collected from the western (mainly from Wyoming) and eastern United States (mainly along the Appalachian Mountains). The western United States data were previously collected from 35 individuals (20 males, 15 females) that were mostly year‐round residents (data collected by TAM and TEK). The eastern United States data were from 9 individuals (4 males, 5 females) that migrated to and from the summering grounds in Canada and wintering grounds in the eastern United States (data collected by the Eastern Golden Eagle Working Group; http://egewg.org/).

### 2.2 Study Regions

The western and eastern United States regions considered in this work captured a wide range of topographical and climatic conditions. As per the Level‐I ecoregion classification of North America (Omernik and Griffith 2014), the western United States data were from the ecoregions of Great Plains and North American Deserts, while the eastern United States data were from the ecoregion of Eastern Temperate Forests. According to the turbine location data from the US Wind Turbine Database (Hoen et al. 2018), the western and eastern data overlapped with about 2200 and 1600 operational turbines, respectively. This overlap between wind energy development and golden eagle habitat made these regions desirable and suitable for this study.

### 2.3 Telemetry Data

The telemetry data were collected using solar‐powered global positioning system (GPS) units from Cellular Tracking Technologies (Cape May, NJ, USA) using a backpack harness (Kenward 1985). The units collected time‐stamped data including latitude, longitude, and altitude above geoid (mean sea level) at 3 s–15 min intervals (variable sampling rate). More details on data collection and management can be found elsewhere (Miller et al. 2014; Katzner, Smith, et al. 2012; Katzner, Brandes, et al. 2012; Duerr et al. 2015; Bohrer et al. 2012; Duerr et al. 2019, 2012; Lanzone et al. 2012; Poessel, Duerr, et al. 2018; Poessel, Brandt, et al. 2018).

We performed a series of preprocessing tasks to prepare the telemetry data for model calibration. We first converted the geographic positional data (longitude and latitude) into the North America Albers Equal Area Conic projection to obtain the horizontal position of the bird in meters. We then eliminated those data with abrupt and unrealistic temporal changes in altitude by ensuring that the maximum temporal change in vertical speed is less than 6 m/s^2^. We chose this threshold to ensure extreme gliding and soaring vertical movements are included in the calibration data. We also eliminated the data that corresponded to horizontal speeds less than 1 m/s since we were primarily focused on modeling flighted movements and low horizontal speeds are mostly stationary.

Next, we segmented the processed telemetry data into individual tracks using the following three rules: (1) the time interval between sequential data points within each track must not be more than 10 s, (2) the total time duration of each track must be more than 5 min, and (3) each track must contain at least 50 data points. The resulting data are referred to as *track data*. The first rule ensured that the movements within each track were captured at high temporal resolutions to allow for accurate resampling of the track at 1‐s resolution. The second rule ensured that the persistent movements were captured in each track to allow for modeling the temporal correlations in movement dynamics. The third rule ensured that the average time interval within each track was no more than 6 s.

### 2.4 Track Resampling

Each telemetry track contained movement sampled at variable time intervals with the median time interval of 6 s. For initial data exploration and subsequent movement modeling, movement data must be at a constant time interval. We chose this interval to be 1 s to ensure adequate capturing of fine scale maneuvers. Inclusion of GPS errors in track resampling is required at such a fine temporal scale, whereas these errors can be safely ignored when modeling hourly scale or daily‐scale movements. Standard cubic spline interpolation operates by ignoring GPS errors, and even a small error in positional observations can propagate to produce unrealistic estimates of higher‐order movement variables (Péron et al. 2020). While measurement‐error accounting spline methods have been developed (Buderman et al. 2016), we chose the Kalman smoother approach for its robust framework for incorporating observation uncertainties and propagating these uncertainties to derived movement variables.

We used the Kalman filtering approach that uses a Kalman smoother to handle positional errors and produce estimates for the mean and variance (uncertainty) for position, velocity, acceleration, and the rate of change of heading (hereafter referred to as heading rate) at each time step (Särkkä 2013). Table 1 lists the movement variables computed by the Kalman smoother for each telemetry track. Appendix A details the Kalman smoother, including its mathematical details, numerical implementation, and contrast between the Kalman filter and the cubic spline interpolation.

**TABLE 1:** Movement and environmental variables available at 1‐s intervals following track resampling and data annotation.

Variable	Notation	Unit	Source	Comments
*Movement dynamics*				
Horizontal position	$p_{x}$, $p_{y}$	m	Kalman smoother^a^	+ve towards east ($p_{x}$), north ($p_{y}$)
Altitude	$p_{z}$	m	Kalman smoother^a^	Above mean sea level
Velocities	$v_{x}$, $v_{y}$, $v_{z}$	m/s	Kalman smoother^a^	Using $p_{x}$, $p_{y}$, and $p_{z}$
Accelerations	$a_{x}$, $a_{y}$, $a_{z}$	m/s 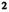	Kalman smoother^a^	Using $p_{x}$, $p_{y}$, and $p_{z}$
Horizontal speed	$v_{h}$	m/s	${\left(v_{x}^{2}+v_{y}^{2}\right)}^{1/2}$	Using $v_{x}$ and $v_{y}$
Heading	θ	deg	$\arctan\left(v_{x}/v_{y}\right)$	+ve clockwise, $0^{o}$ = North
Heading rate	ω	deg/s	$\left(a_{x}v_{y}-a_{y}v_{x}\right)/v_{h}^{2}$	+ve clockwise
*Environmental conditions*				
Ground elevation	$h_{g}$	m	3DEP^b^	Above mean sea level
Ground slope	$\phi_{s}$	deg	3DEP^b^	$\left[0,90\right)$, $0^{o}$ = Flat‐area
Ground aspect	$\phi_{a}$	deg	3DEP^b^	$\left(-180,180\right)$, $0^{o}$ = Northerly slopes
Altitude AGL	$h_{\mathrm{agl}}$	m	$p_{z}-h_{g}$	
Wind speed	w	m/s	HRRR^c^	80 m AGL
Wind direction	$\phi_{w}$	deg	HRRR^c^	80 m AGL, $0^{o}$ = Northerly wind
Orographic updraft	$w_{o}$	m/s	$w\sin\left(\phi_{s}\right)\cos\left(\phi_{w}-\phi_{a}\right)$	Brandes and Ombalski (2004)
Tailwind speed	$w_{t}$	m/s	$w\cos\left(\theta -\phi_{w}\right)$	wind ∥ to heading (θ)
Crosswind speed	$w_{c}$	m/s	$w\sin\left(\theta -\phi_{w}\right)$	wind ⊥ to heading (θ)

^a^ Executed at 1‐Hz resolution on each telemetry track.

^b^ Using bilinear interpolation on 3DEP's 10‐m resolution data.

^c^ Using bilinear interpolation on HRRR's 3‐km resolution data.

### 2.5 Data Annotation

We used the digital elevation model (DEM) from the US Geological Survey's 3D Elevation Program (3DEP) to annotate the track data with terrain properties (US Geological Survey 2022). The elevation, slope, and aspect of the ground are available from 3DEP at a uniform resolution of 1/3 arcsecond (approximately 10 m) for the continental United States. We used bilinear interpolation on the 10‐m resolution 3DEP grid to annotate elevation, slope, and aspect values to each location in the track data.

We imported wind conditions from the National Oceanic and Atmospheric Administration's High‐Resolution Rapid Refresh (HRRR) data set (Benjamin 2016). The HRRR model is based on the established Weather Research and Forecasting numerical weather prediction model (Skamarock et al. 2019) and has a uniform 3 km resolution grid that covers the continental United States. We used the instantaneous 1‐h HRRR data at 80‐m altitude AGL for annotating the wind speed and direction to the track data by using linear interpolation in time and bilinear interpolation in the horizontal plane. The variation in wind conditions over altitude AGL was not considered, since we eventually only included data within RSZ for model calibration.

We computed an estimate of the orographic updraft using the wind conditions and the terrain properties from 3DEP (Brandes and Ombalski 2004) (details in Table 1). This estimation computed only the updrafts; the downdrafts (negative values of updraft) were set to zero as our model focuses on eagle attraction to favorable soaring conditions rather than avoidance of unfavorable regions. We then smoothed the estimated updraft field by passing it through a Gaussian filter with a kernel radius of 250 m (from Python's SciPy library (Virtanen et al. 2020)). This smoothing ensured that the updraft values were spatially correlated and that the updraft field was free of abrupt variations resulting from the crude estimation.

### 2.6 Movement Model

We developed a discrete‐time first‐order Markov process to model the following three movement quantities (hereby referred to as predictors): (1) vertical speed, $v_{z}$, (2) horizontal speed, $v_{h}$, and (3) heading rate, ω. We chose to model the heading rate rather than heading itself for the following reasons: (1) heading is a circular variable ($0^{o}$ to $360^{o}$) that requires special statistical treatment and (2) heading rate could be naturally related to gradients in surrounding conditions specifically for finescale movement modeling. A 3D path can be generated given the value of these predictors at each simulation time step by following these steps: (1) compute the heading by integrating the heading rate over the time step and (2) use the updated heading with horizontal speed to compute new horizontal positions. The predictors at time $t_{k+1}$ are modeled as a linear function of all the ecological covariates at the previous time $t_{k}$. A given predictor $y_{k+1}$ at time $t_{k+1}$ is modeled as a sum of a *mean model* and an *error model*. Mathematically speaking,

(1) $$y_{k+1}=\underset{\text{Mean}\;\text{model}}{\underbrace{\sum_{j=1}^{N_{\psi }}\beta_{j}^{\text{mean}}\psi_{j,k}}}+\epsilon_{k};\;\epsilon_{k}∼\underset{\text{Error}\;\text{model}}{\underbrace{N\left(0,{\left(\sum_{j=1}^{N_{\psi }}\beta_{j}^{\text{error}}\psi_{j,k}\right)}^{2}\right)}},$$

where $N_{\psi }$ is the number of covariates, $\beta_{j}^{\text{mean}}$ is the unknown weight capturing the effect of covariate $\psi_{j,k}$ within the mean model, $\psi_{j,k}$ is the j 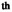 covariate at time $t_{k}$, $\epsilon_{k}$ is the model error capturing the discrepancy between the data and mean model, $N\left(\mu ,\sigma^{2}\right)$ is a Gaussian distribution with mean μ and strength σ, and $\beta_{j}^{\text{error}}$ is the unknown weight capturing the effect of covariate $\psi_{j,k}$ within the error model. The mean model captures the mean behavioral response, while the error model captures the deviations away from the mean response, as represented in the track telemetry data. We first fitted the mean model to the track data to compute the weights $\beta_{j}^{\text{mean}}$ by least squares estimation. The model error $\epsilon_{k}$ was computed by subtracting the fitted mean model from the telemetry data. The square of this error was then used to fit the error model and compute the weights $\beta_{j}^{\text{error}}$ of the error model.

At minimum, we included the following covariates for the three predictors: (1) crosswind speed, (2) tailwind speed, (3) orographic updraft speed, and (4) altitude AGL. The tailwind and crosswind components of the wind were included to quantify the influence of the relative wind on the fine‐scale changes in movement dynamics. The time‐lagged versions of each predictor at lags of 5 s, 10 s and 30 s were also included as covariates to capture the temporal correlation in predictors. We chose the same ecological covariates for both the mean and error models of a given predictor at time $t_{k+1}$. Given the primary focus of this work is on fine‐scale movement modeling within the RSZ, we only considered the track data below 200 m AGL for model calibration.

### 2.7 Directional Intent

**FIGURE 1:**
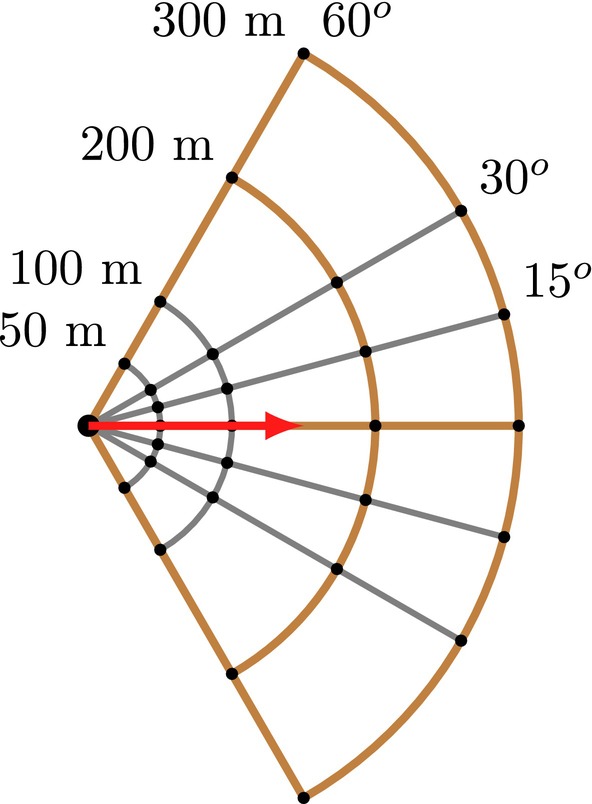
Look‐ahead region. The arrow represents the current heading.

Incorporating the effect of environmental conditions away from the current position is critical for modeling the fine‐scale movement choices of raptors within RSZ. In this work, we attempted to quantify the relationship between the heading rate and the updraft conditions in the region directly ahead in the immediate direction of travel. We hereby refer to this region as the *look‐ahead* region. Figure 1 shows the 28 locations we chose to represent the look‐ahead region. Each look‐ahead location is denoted by the pair (α,d), where α (deg) is the angle away from the immediate direction of movement (i.e., heading θ) and d (m) is the radial distance away from the current position. α and d are hereby referred to as the look‐ahead angle and the look‐angle distance, respectively. We considered α values of ±15, ±30, and ±60 degrees and d values of 50, 100, 200, and 300 m.

We considered the orographic updraft $w_{o}$ as a potential covariate within the look‐ahead region. $w_{o}$ varies significantly at the spatial scales considered within the look‐ahead region and was available at 10‐m spatial resolution from 3DEP data. We annotated $w_{o}$ at the 28 look‐ahead locations for each data point in the track data. Since we only considered wind conditions at 80‐m altitude AGL for computing $w_{o}$, we assumed that the eagles experience these updrafts irrespective of their altitude AGL. Wind speed and direction do not vary significantly at these spatial scales so we did not consider those within the look‐ahead region.

We started by computing the orographic updraft value $w_{o}^{\left(\alpha ,d\right)}$ at the 28 look‐ahead points shown in Figure 1. Then, we computed the difference $\Delta w_{o}^{\left(\alpha ,d\right)}=w_{o}^{\left(\alpha ,d\right)}-w_{o}^{\left(0,d\right)}$ for the 24 look‐ahead points with $\alpha \neq 0^{o}$. If $\Delta w_{o}^{\left(\alpha ,d\right)}$ is positive, an eagle may attempt to catch this higher updraft region around location $\left(\alpha ,d\right)$, rather than heading in the same direction towards $\left(0,d\right)$. A brute‐force modeling approach would have involved inserting these 28 values of $\Delta w_{o}^{\left(\alpha ,d\right)}$ as individual covariates within the Markov model for the heading rate. However, these 56 covariates would most likely be correlated with each other and would potentially include repeated information about the underlying terrain and wind conditions. Moreover, the resulting predictive model will be linked to the location of these 28 look‐ahead locations, meaning an end‐user of the model needs to have orographic updraft and terrain elevation available at these exact locations. This approach would introduce inconvenient rigidity into the predictive model and will hinder the broader appeal of the resulting tool.

To avoid this issue, we undertook the following model discovery approach to find an empirical relation between heading rate (ω), updraft difference $\Delta w_{o}^{\left(\alpha ,d\right)}$, look‐ahead angle α, and look‐ahead distance d. This approach aimed to collapse the 24 look‐ahead locations into a single generalizable equation applicable to any updraft field. Once this relationship is discovered, it could potentially be applicable to other eagles not represented in the training telemetry data. First, we partitioned the $\Delta w_{o}^{\left(\alpha ,d\right)}$ values into several bins spanning its 1st and 99th percentile. Given a minimum and maximum value of $\Delta w_{o}^{\left(\alpha ,d\right)}$ for each bin, we gathered the corresponding data for ω. We then randomly sampled a fixed amount of points from these ω values. A limited amount of telemetry data was available for high $\Delta w_{o}^{\left(\alpha ,d\right)}$ values and this sampling approach ensured that the data in each bin are represented equally. We then plotted the median of this randomly sampled ω data for various bins of $\Delta w_{o}^{\left(\alpha ,d\right)}$. Through trial‐and‐error curve fitting, we determined how the relationship between ω and $\Delta w_{o}^{\left(\alpha ,d\right)}$ varied with α and d. We also investigated the relation of the standard deviation of heading rate with varying α, d and $\Delta w_{o}^{\left(\alpha ,d\right)}$.

### 2.8 Model Validation

It is important to note that the model calibration (Section 2.6) used track‐anonymized data, where individual time steps from all tracks were pooled without maintaining track sequence or continuity. Thus, the model calibration focused on instantaneous relationships between predictors and covariates. In contrast, model validation requires continuous track sequences to assess the model's ability to predict sequential movements over time. While individual data points from validation tracks may appear in the calibration dataset, the validation specifically tests the model's capability to generate realistic continuous trajectories, which was not explicitly part of the calibration process.

We randomly selected 200 tracks of 3‐min duration from the track data to validate the calibrated movement model. We imposed the condition that at least 75% of each selected track (or 75% of 3*60 data points) needed to have an altitude AGL of less than 200 m (i.e., within the RSZ). We imposed this condition to ensure that these validation tracks contain data similar to those used in calibrating the Markov models of predictors. We then imported the terrain properties from 3DEP for a rectangular region that contained the validation track and whose boundaries were at minimum 2 km away from each point in the validation track. This rectangular region is hereby referred to as the *domain*. The inclusion of additional terrain around the track ensured that the simulated paths were able to explore regions not traversed by the validation track. The wind conditions and the orographic updrafts within this domain were obtained using the same approach used in data annotation (Section 2.5).

For each validation track, we simulated 100 paths using the calibrated model with the initial movement state randomly sampled from the probability distribution (with the Kalman smoother) corresponding to the start of the validation track. At each time step $t_{k}$ of the simulation, the predictors ($v_{h}$, $v_{z}$, and ω) were computed using calibrated Markov models based on the look‐ahead conditions at the current location. Subsequently, we simulated the 3D path ($p_{x}$, $p_{y}$, $p_{z}$) using

(2) $${\left\{\begin{array}{c} p_{x}\\ p_{y}\\ p_{z}\\ \theta \end{array}\right\}}_{k+1}={\left\{\begin{array}{c} p_{x}\\ p_{y}\\ p_{z}\\ \theta \end{array}\right\}}_{k}+T{\left\{\begin{array}{c} v_{h}\sin\left(\theta \right)\\ v_{h}\cos\left(\theta \right)\\ v_{z}\\ \omega \end{array}\right\}}_{k},$$

where time interval T was chosen as 1‐s (similar to track data). We simulated each path for a total duration of 3 min, similar to the length of validation tracks. Since the Markov model is stochastic in nature (through the error model; Equation 1), each simulated path is expected to have different movement characteristics for a given domain and static environmental conditions (orographic updraft field and wind conditions). We terminated the simulation when one of the points in the look‐ahead region fell outside the domain for that particular path.

Given the time history of a simulated path, we computed the radial distance between the 3D positions from the simulated path and the validation track at each time step. This radial distance was computed across all simulated paths and across all validation tracks. The resulting probability distribution of this radial distance at varying times (from the start of the track) provided a quantitative indicator of the predictive performance of the calibrated model. We particularly focused on the 2nd quartile (50th percentile) of this distribution. We also converted the radial distances to a representative number of rotor diameters, assuming a 130‐m rotor diameter for a typical land‐based wind turbine (Bortolotti et al. 2019). This quantity was intended to contrast the model performance within the context of real‐time turbine curtailment solutions as rotor diameter plays a key role in determining turbine curtailment strategies (McClure et al. 2021).

We contrasted the model performance against a constant velocity approach wherein an eagle is assumed to keep traveling with the heading and velocity corresponding to the start of the validation track. Such an approach is often used to predict the potential conflict of flighted eagles with rotating wind turbines where the starting location of an approaching eagle is available using sensor‐based automated detection technologies (McClure et al. 2018, 2021). We also compared the model predictive performance when the validation data satisfied the following flight conditions: (1) wind speed was ≥ 5 m/s, (2) altitude AGL was within RSZ, and (3) orographic updraft was ≥ 0.75 m/s. We applied these conditions to the validation data in a cumulative manner. We particularly focused on the time range of 2.5 min to 3 min from the start of the validation track to contrast the model performance across all these conditions.

### 2.9 Collision Risk

We used the 100 simulated paths corresponding to each validation track to compute the eagle counts at the uniformly gridded domain. We associated each location on the simulated path location with a unique grid point in the domain through a nearest‐neighbor approach. We then applied a Gaussian filter (kernel radius of 250 m) to these grid‐based eagle counts to obtain a smooth presence density of model‐simulated eagle flight paths. We only used the simulated locations where the altitude AGL was within the RSZ to capture the turbine collision risk. We then normalized the resulting presence density to ensure it varied between zero and one, hereby referred to as the *collision risk map*. We computed the collision risk map for varying time lags from the start of the validation track. This model‐simulated time‐explicit risk map provides a relative indication of locations/turbines in the domain with the most potential for collision risk. A value of one implied the most risk, while a value of zero implied the lowest risk, for the given domain and wind conditions.

## 3 Results

### 3.1 Data Processing

Using the track identification rules, we identified 7411 tracks in the western USA data and 607 tracks in the eastern US data (Table 2). This constitutes about 60% and 25% of the raw telemetry data we had from the western and eastern United States, respectively (Table 2). Considering only the track data, we found that about 80% of the western US data was sampled under or at 6‐s intervals, while the eastern US data had only 25% data under or at 6‐s intervals. We found that about 25% of the tracks in the western US data were longer than 30‐min, while only 15% were longer than 30‐min for the eastern US data. Other important properties of the telemetry data are listed in Table 2.

**TABLE 2:** Properties of the golden eagle telemetry data.

	Point count (Raw data)	% of data as tracks^a^	Time range (Track data)	Eagle count (Track data)	% of track data in RSZ^b^
Eastern US	594,760	24% (607)	03/2016–12/2020	9	48% (102 h)
Western US	3,556,385	60% (7411)	03/2019–05/2020	35	41% (1332 h)

^a^ Value in brackets is the number of tracks.

^b^ Value in brackets is the total duration of track data within RSZ, at 1‐s resolution.

**FIGURE 2:**
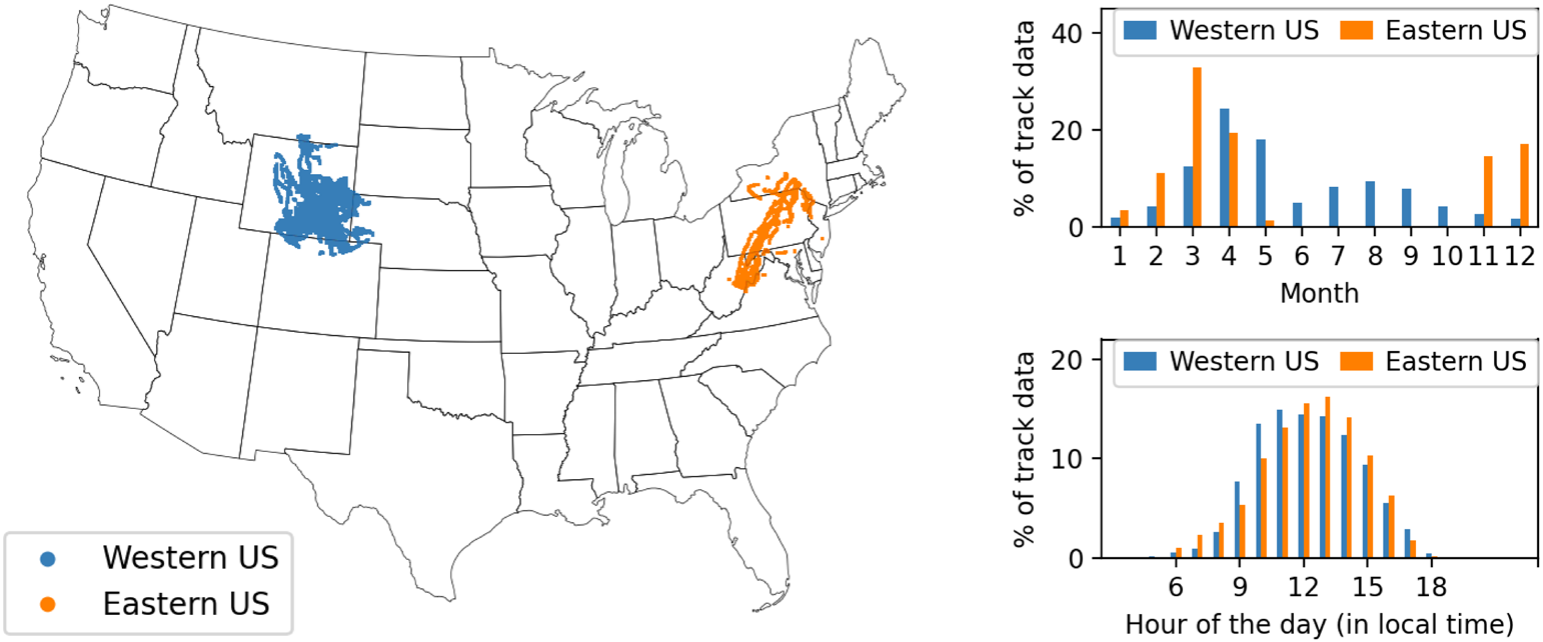
Track telemetry track data considered for movement modeling.

The majority of the telemetry data were from the spring season for both the western and eastern United States (Figure 2). Limited data were available in the summer and autumn seasons for both the western and eastern United States. We found that significant eastern US data were available across the winter season. Most of the track data were available during the daytime, peaking at around noon time for both eastern and western United States (Figure 2).

**FIGURE A4:**
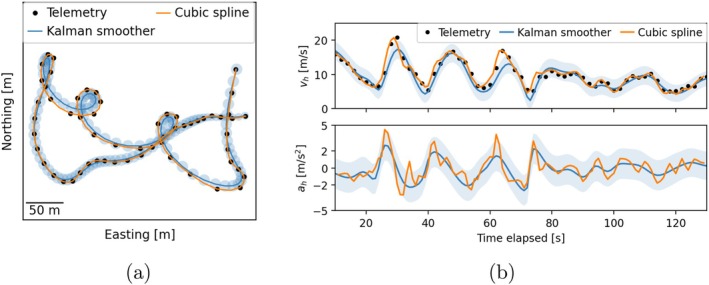
Comparison of Kalman smoother and cubic spline interpolation for a 2‐min segment of a sample telemetry track. (a) Horizontal path, and (b) time history of horizontal velocity, $v_{h}$, and horizontal acceleration (tangential), $a_{h}$. The instantaneous horizontal velocity available from the telemetry unit is shown in (a).

For track resampling, we found that both cubic spline and Kalman smoother performed comparatively when contrasting the mean resampled horizontal path of a track. However, a significant difference in performance existed for these two techniques when computing higher‐order variables such as $v_{h}$ and $a_{h}$ ($a_{h}$ is used to compute heading rate). We found that the standard cubic spline approach mostly tried to match the positional telemetry data, while the corresponding Kalman smoother estimate tended to capture the general trend in the data while respecting the GPS errors (approximately 2 m horizontally and 6 m vertically; see Appendix A). The Kalman smoother produced more realistic estimates of higher‐order movement variables such as velocity and acceleration (Figure A4b), which are critical for computing heading rate, because it does not attempt to exactly fit noisy observations.

### 3.2 Model Calibration

**FIGURE 3:**
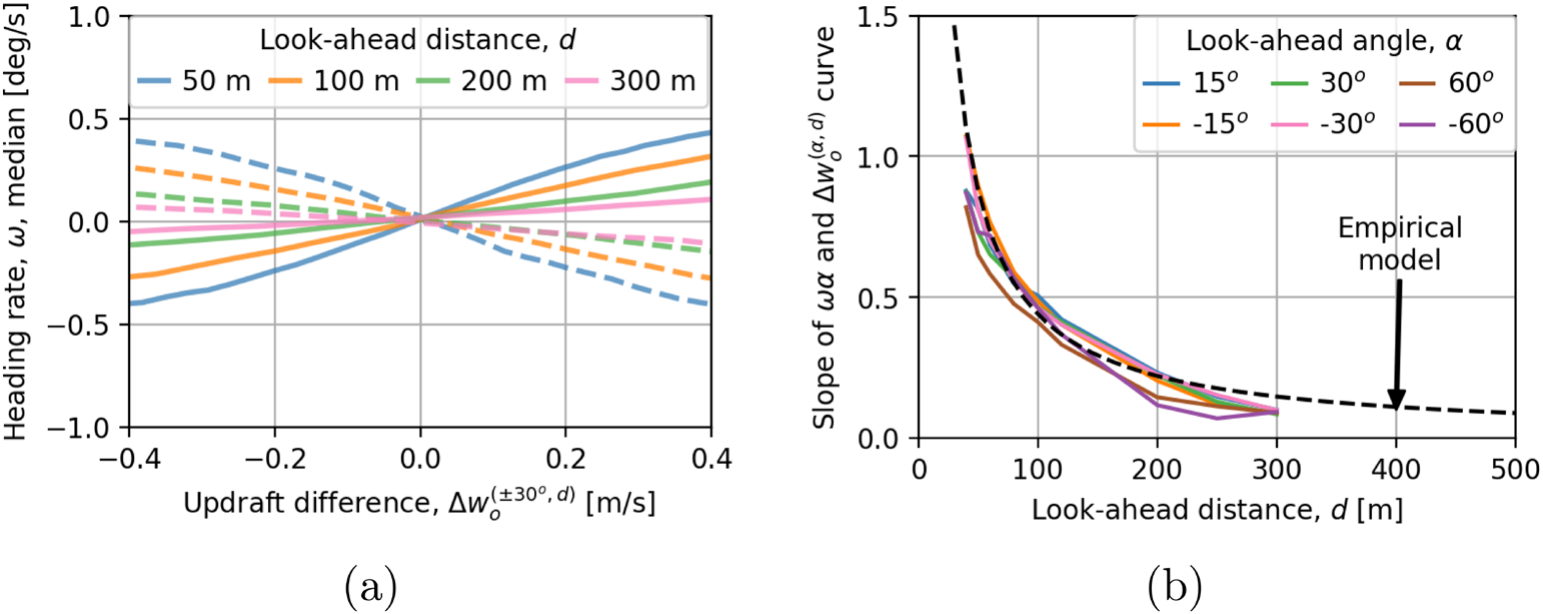
Variation in heading rate with varying values of look‐ahead angle α, look distance d and updraft difference $\Delta w_{o}^{\left(\alpha ,d\right)}$. (a) $\alpha =30^{o}$ case, (b) Predictions from the empirical model. The solid lines in (a) correspond to $+30^{o}$ while the dashed lines correspond to $-30^{o}$.

We found that the median of the heading rate increased linearly with the increasing value of the updraft difference $\Delta w_{o}^{\left(\alpha ,d\right)}$ for a fixed α value of $30^{o}$ and across many values of d (Figure 3a). The heading rate was found to be positive (clockwise turning) when the difference $\Delta w_{o}^{\left(\alpha ,d\right)}$ was positive for $\alpha =30^{o}$, meaning the eagles (on average) were attempting a right turn towards $\left(30,d\right)$ when $w_{o}^{\left(30,d\right)}>w_{o}^{\left(0,d\right)}$. We found that the rate of increase in heading rate with increasing $\Delta w_{o}^{\left(\alpha ,d\right)}$ was higher for lower d values (Figure 3a). We obtained a similar linear relationship between ω and $\Delta w_{o}^{\left(\alpha ,d\right)}$ for various values of α (results not reported here). Given a fixed d, we found that the rate of increase in heading rate with increasing $\Delta w_{o}^{\left(\alpha ,d\right)}$ was higher for lower α values.

The slope of the relationship between ωα and $\Delta w_{o}^{\left(\alpha ,d\right)}$ across various α and d values collapsed to a unique curve (Figure 3b). After trial‐and‐error curve‐fitting, we discovered that this relationship can be captured through the relation

(3) $$\omega =c_{\omega }\frac{\Delta w_{o}^{\left(\alpha ,d\right)}}{d\cdot \alpha }\;\Rightarrow \;\frac{\partial \theta }{\partial t}=\frac{c_{\omega }}{d}\frac{\partial w_{o}^{\left(\alpha ,d\right)}}{\partial \alpha },$$

where $c_{\omega }$ is a parameter that determines how an eagle reacts to a particular α, d, and $\Delta w_{o}^{\left(\alpha ,d\right)}$. Note that the updraft difference $\Delta w_{0}^{\left(\alpha ,d\right)}$ is normalized by the arc length from 0° to α at radial distance d. There was no significant dependence between the standard deviation of heading rate with varying α, d and $\Delta w_{o}^{\left(\alpha ,d\right)}$.

### 3.3 Model Validation

**FIGURE 4:**
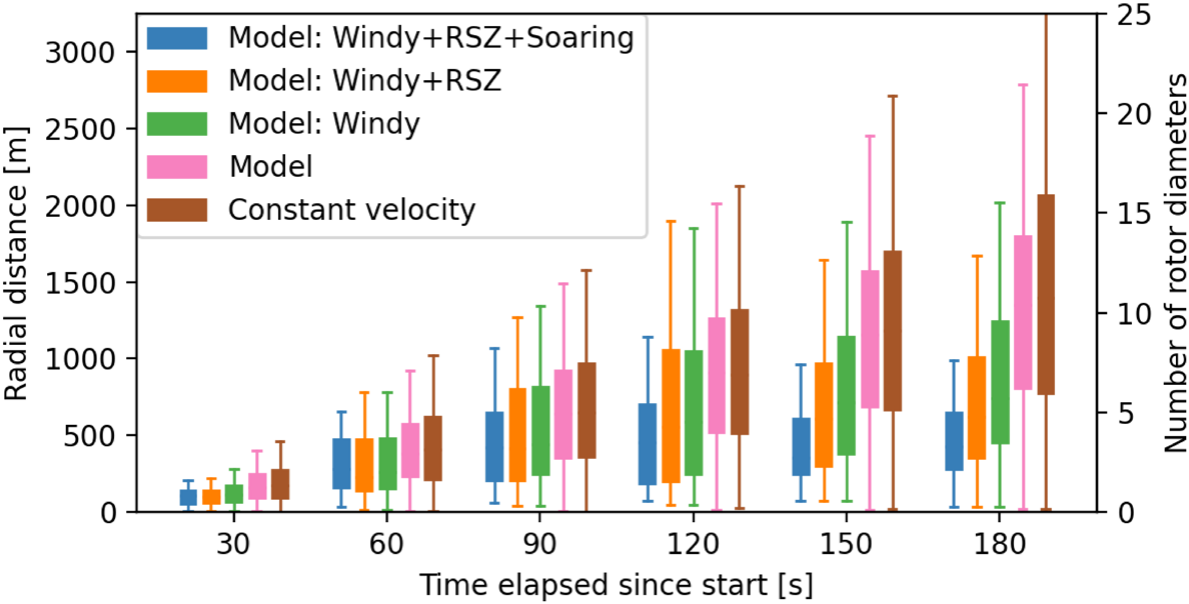
Distribution of radial distance between observed and model‐simulated tracks for varying time lags. Windy = winds greater than 5 m/s, RSZ = altitude AGL less than 200 m, Soaring = orographic updrafts greater than 0.75 m/s.

We found that the model performed marginally better than the constant velocity approach when considering all the validation track data. The lower wind‐speed cutoffs for selecting the track validation data are not shown here for brevity. However, the model performed significantly better when considering only the validation track data where wind speeds were 5 m/s or higher (Windy case in Figure 4). In particular, at 3‐min from the start, about 50% of the time (2nd quartile) the model estimated the predicted location of an eagle within eight rotor diameters (9 D), compared to about 12 D for the constant velocity model. The 2nd quartile (at 3‐min) dropped to 6 D when further focusing on the validation data for which altitude AGL was within RSZ (Windy + RSZ case in Figure 4). Therefore, the model performed about two times better than the constant velocity model when focusing on situations with strong winds (i.e., when turbine blades rotate) and flights within the RSZ. This improvement in model performance was observed for all time lags (Figure 4). The 2nd quartile (at 3‐min) further dropped to 4 D when considering the validation data where orographic updrafts were greater than 0.75 m/s (Windy + RSZ + Soaring case in Figure 4). Therefore, the model performed about three times better than the constant velocity model for predicting golden eagle flight for up to 3‐min from the start.

**FIGURE 5:**
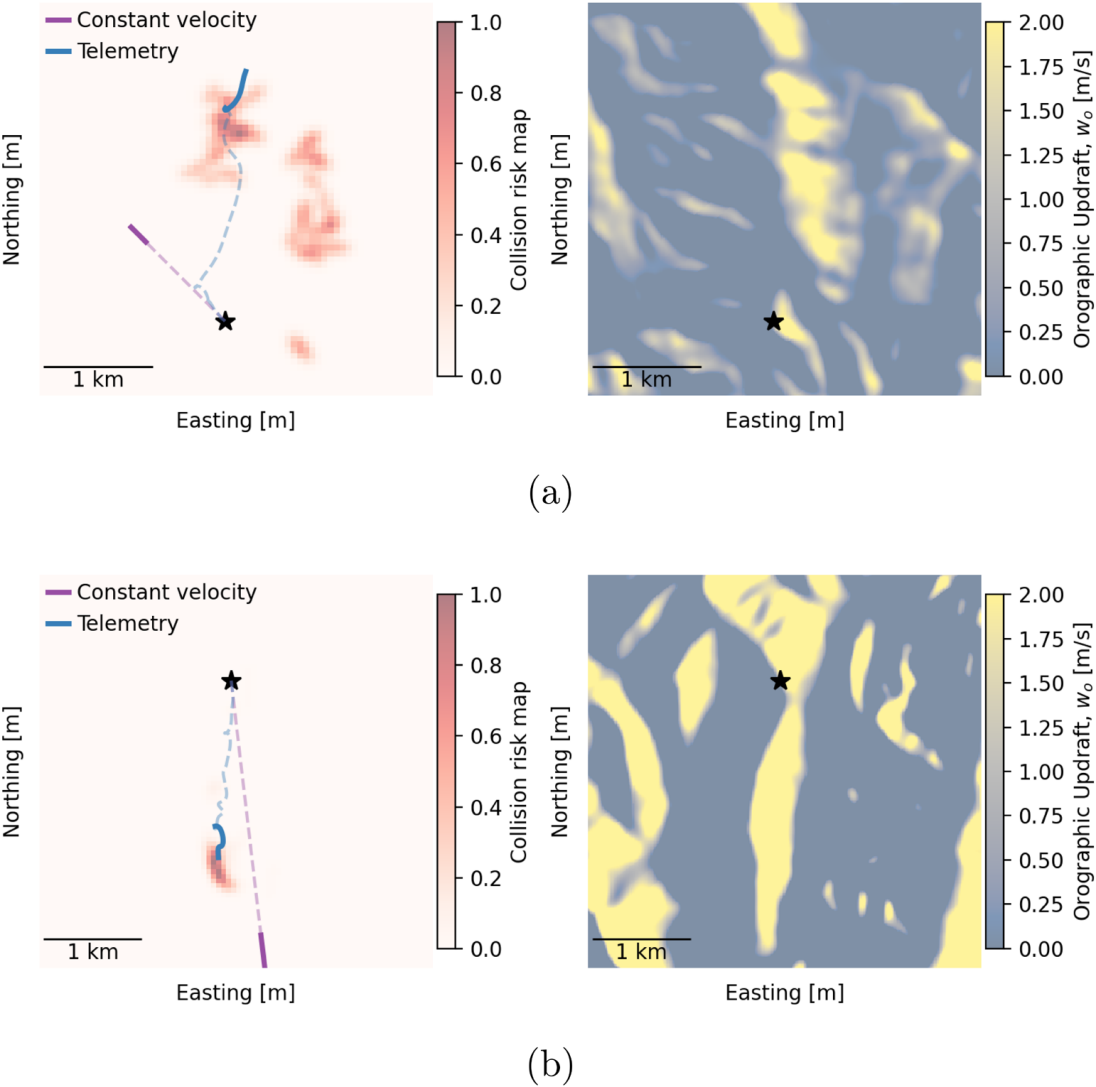
Model‐simulated collision risk map for two selected validation tracks. The dashed and solid lines represent the time range 0–2.5 min and 2.5–3 min, respectively. The starred point shows the starting location.

Figure 5 shows the model‐simulated collision risk map for two selected validated tracks, along with the corresponding orographic updraft field. The model reasonably predicted the location of tagged eagles at 3 min from the start for both cases (Figure 5). For track (a), unlike the constant velocity approach, the model was able to capture the sharp turn towards a higher orographic updraft region (Figure 5a). For track (b), the model predicted that the simulated eagles soared on the west side of the north–south ridgeline given strong westerly winds, while the constant velocity model inaccurately predicted eagles traveling to the leeward side of the ridge where there were limited orographic updrafts (Figure 5b).

## 4 Discussion and Conclusion

Technology for minimizing raptor fatalities at wind energy facilities can benefit from advances in predictive movement modeling and automated risk mitigation methods. Of particular interest are species‐specific models that can be utilized in real‐time, require only readily available movement and environmental data, take into account updraft conditions in an eagle's line‐of‐sight, and can predict low‐altitude movements at fine‐scale temporal resolutions with reasonable accuracy. In this work, we designed, calibrated, and validated a data‐driven agent‐based movement model for golden eagles within the United States that possesses all of these capabilities and is capable of aiding turbine curtailment technologies in further minimizing raptor fatalities at wind plants. This study was motivated by our previous work in predictive modeling of transiting golden eagle movements, such as migration, but was primarily focused on real‐time predictions under situations where latent eagle‐specific variables such as preferred direction of movement are not known in advance (Sandhu et al. 2022). The proposed modeling framework is general (in terms of acceptable environmental and telemetry data inputs) and can be applied to different species of soaring birds for which there is a known risk of collision with turbines or other infrastructure.

### 4.1 Telemetry Data

By using telemetry data from 44 individuals, tracked over multiple seasons/years and two different ecoregions, we created a calibrated model that could be applied to a wide range of regions within the United States and beyond (Omernik and Griffith 2014). Segmenting the telemetry data into various specific situations (i.e., by season or time of the day) might provide greater insight into the eagle behavior but may not have resulted in a robust predictive movement model. This choice of telemetry data was also motivated by the presence of a high number of operational turbines overlapping the two regions, also highlighting the potential risk of turbine collision to the golden eagle population in these regions.

The segmentation of the telemetry data into tracks containing movements captured at high temporal resolutions enabled fine‐scale flight behaviors to be well‐captured in the tracks. Shorter duration of tracks in the eastern data could be due to the higher sampling interval of some telemetry units used for the eastern golden eagles. This higher time interval could also explain the lower percentage of raw telemetry data being identified into tracks for the eastern data. Alternatively, eagles may behave differently due to ecological differences between the two regions. For example, eagles in the western United States, that occupy more open landscapes (e.g., sagebrush and grasslands) may engage more in flighted hinting, whereas eagles in the eastern United States, that primarily occupy forested areas (Miller et al. 2017), may engage in more perch hunting. The higher percentage of track data within RSZ for the eastern data could be explained by the widespread availability of orographic updrafts (leading to low‐altitude flight) in the Appalachian Mountains or the times of years when the data were collected there, i.e., during cooler months when thermal updrafts are weaker and less available. Conversely, a considerable portion of the track data within the western data was collected during warmer months when thermals are stronger and more available, which may explain the smaller percentage of track data within RSZ for the western data.

### 4.2 Data Processing

We designed the data processing pipeline for the golden eagle telemetry data so that it could potentially be extended to prepare track‐based model calibration data from other avian species. The removal of abrupt altitude variations from the telemetry data ensured that altitude AGL is accurately captured in the calibration data. Typically, altitude (vertical) measurements by GPS have higher errors than horizontal position; when calculating altitude AGL, these errors can be compounded by errors in horizontal position and DEM errors (Poessel, Duerr, et al. 2018; Poessel, Brandt, et al. 2018). Also, the bilinear interpolation of ground elevation from a 10‐m resolution grid to the exact eagle position can further exacerbate these errors, especially around ridges. Note that along and above ridges are where most orographic soaring occurs and, coincidentally, also where most turbines are positioned. Due to this unique situation around ridges, we did obtain negative values of altitude AGL and we did decide to include those in model calibration. Therefore, the predictive model of vertical movements ended up simulating occasional instances of negative altitude AGL as well. These negative altitude AGL movements were considered within RSZ and were included in the computation of collision risk maps.

Resampling through Kalman smoothing enabled inclusion of GPS errors when reconstructing fine‐scale movements at 1 s intervals. The superior performance of the Kalman smoother over spline interpolation techniques for capturing higher‐order dynamic variables demonstrated the influence of positional errors on velocities and accelerations, which are the key variables in the proposed model. The uncertainties in the resampled dynamic state of the eagle provided random initial conditions for the simulated paths during model validation. In the predictive mode, these uncertainties could potentially be derived from sensor‐based avian detection systems.

The availability of terrain features at 10‐m resolution from 3DEP played a key role in the design of the model structure and our choice of 1‐s as the temporal scale. Lower resolution terrain data would have resulted in a coarser representation of the orographic updraft field within the look‐ahead region, which could have hindered the discovery of the empirical model between heading rate and look‐ahead updrafts. The wind conditions did not vary significantly at the spatial scales of look‐ahead region, and therefore, a 3 km resolution (HRRR) was sufficient for this study.

### 4.3 Eagle Flight Behavior

Low‐altitude flight of golden eagles is highly influenced by the topography and the orographic updraft availability in their vicinity (Duerr et al. 2019). Irrespective of internal behavioral state (hunting/migrating/perching), a golden eagle might scan the underlying terrain and make predictions on potential updraft availability at locations it can visibly see. Such decision‐making can then lead to directional decisions that may be contradictory to the updraft conditions at the current position. Inclusion of look‐ahead region was a key novelty of the proposed model which differentiated this model from other agent‐based models of raptors that solely rely on covariates at the current location (Eisaguirre et al. 2019; Sur et al. 2021). Using the empirical relation in Equation (3), we replaced the 24 updraft covariates $w_{o}^{\left(\alpha ,d\right)}$ with a single covariate $\Delta w_{o}^{\left(\alpha ,d\right)}/\left(d\cdot \alpha \right)$ in the Markov model for heading rate ω. The higher rate of increase in heading rate for shorter look‐ahead distances demonstrated that the eagles responded more to a higher updraft availability closer to their current location than those far away. Based on the telemetry data, we found a sharp decline in the response of golden eagles to orographic updrafts beyond the radial distance of 200‐m. Meanwhile, the higher rate of increase in heading rate for smaller look‐ahead angles (i.e., narrower look‐ahead sectors) demonstrated that the eagles responded more to higher updraft availability in regions that do not require an eagle to make large changes in their heading.

This discovery of a well‐defined empirical relation between heading rate and the orographic updrafts within the look‐ahead region highlighted the dependence of directional decision‐making on conditions away from the eagle's current location. Moreover, the calibrated model can now be applied for any look‐ahead updraft field through $w_{o}^{\left(\alpha ,d\right)}$ in Equation (3), which basically decoupled the setup for the look‐ahead region between calibration and prediction steps. The effect of d and α tapered beyond 300 m and ±60 deg., respectively, and so we did not consider values beyond these limits in the model calibration. The presence of α and d in the denominator in Equation (3) highlighted this tapering. We did not notice a significant relation between the standard deviation of heading rate with varying α, d and $\Delta w_{o}^{\left(\alpha ,d\right)}$ and, therefore, no covariate related to look‐ahead updrafts was considered in the error model for ω. Also, we did not notice a significant relation between the look‐ahead updrafts and the other predictors (vertical velocity and horizontal acceleration) and, therefore, we excluded the look‐ahead conditions from the Markov model of these predictors.

### 4.4 Model Validation

The calibrated model significantly outperformed the constant velocity approach for predicting the location of an approaching eagle up to 3‐min from the start. The enhanced model performance for wind speeds greater than 5 m/s highlighted the superiority of the model in situations where wind turbines are likely to be operational. This model behavior can be explained by the notion that under limited winds/updrafts, fine‐scale eagle movements are potentially dictated by an individual's internal state rather than the current environmental conditions. The increase in model performance for predicting movements with strong winds within the RSZ is attributed to having a stronger orographic updraft field to support low‐altitude flights and highlights the model utility in situations involving a higher risk of turbine collision. The degradation in model predictive performance for lower wind speed cutoff can be attributed to the notion that the orographic updrafts are less prevalent at lower wind speeds and the model capability is limited under such situations.

### 4.5 Application

The proposed model provides a reasonable alternative to existing real‐time path prediction tools utilized in automated turbine curtailment solutions. A simulation domain could be chosen to encompass all turbines in a given wind farm. Once an approaching eagle is detected, the proposed model would be initiated from the detected location with some predefined uncertainty on the initial state of the movement variables. The corresponding wind conditions could potentially be imported from the real‐time data collected by wind turbines and/or local meteorological instruments. Given the starting location and the wind conditions within the domain, the model can simulate multiple paths of the detected eagle. These paths could be combined to obtain a microscale collision risk map that identifies likely turbines in conflict with the approaching eagle. This prediction process could be run in an iterative manner, wherein each update in the detected eagle location would be used to re‐initialize and re‐run the model to produce an updated real‐time collision risk map. These time‐varying risk maps could then inform the curtailment strategy such that the control of curtailed turbines is updated with changes in eagle location. Ultimately, having accurate real‐time collision risk maps can increase safety for eagles and reduce the number of unnecessary shutdowns for wind‐farm operators.

### 4.6 Potential Improvements

The model could potentially be improved in a number of ways. The assumption that eagles in low‐altitude AGL flight experience the same orographic updraft field irrespective of altitude could be relaxed. A more sophisticated orographic updraft model could be included, considering the limitations of the estimation approach employed in this study (Brandes and Ombalski 2004). Two primary considerations are: (1) the updraft estimate is directly proportional to the wind speed, which neglects the decay in orographic updraft with altitude AGL; and (2) the estimated updraft field depends on having a wind‐speed measurement at a particular altitude of interest, which in general is not available for arbitrary movement tracks. A comprehensive discussion of updraft modeling limitations, along with a proposed improved engineering updraft model, can be found in Thedin et al. (2023). In addition to addressing the aforementioned issues, that model also accounts for terrain complexity and non‐local terrain features.

The inclusion of thermal updrafts as a covariate could potentially improve the model performance in predicting high‐altitude movements (Katzner, Smith, et al. 2012; Katzner, Brandes, et al. 2012). We note that the proposed approach does not explicitly quantify the spatial structure of thermal updrafts or the characteristic circling behavior eagles exhibit when using thermal updrafts. Thermal updrafts create localized, transient columns of rising air that differ fundamentally from the persistent, terrain‐linked orographic updrafts our model explicitly represents. However, thermal updrafts often lead to high‐altitude movements (well above RSZ), thereby posing a lower risk of turbine collision. Because the focus of this work is on predicting the risk of turbine collision, we intentionally left out thermal updrafts from the model. Nonetheless, the inclusion of thermal updrafts and its influence on the collision risk maps will be explored in future work.

We anticipate that the model could predict long‐term movements (longer than 3‐min); however, we expect that the model performance will degrade with increasing time difference between the start and prediction times. The predictive performance of the model could be enhanced by specifying a preferred direction of movement, assuming it remains constant throughout the long‐term flight. We anticipate releasing a modified version of the model in the future wherein a preferred direction of movement (such as during migration) could be included in the model structure.

The empirical relation among the heading rate and the look‐ahead updrafts could potentially be extended to other raptor species with adjustments made to the fixed parameter $c_{\omega }$ in Equation (3). We anticipate $c_{\omega }$ to be a function of age/sex/species of an individual raptor. We plan on investigating this relation in future studies.

## Author Contributions

**Rimple Sandhu:** conceptualization (equal), investigation (lead), methodology (lead), software (equal), validation (equal), visualization (equal), writing – original draft (lead). **Charles Tripp:** conceptualization (equal), methodology (equal), software (equal), supervision (equal), writing – review and editing (equal). **Eliot Quon:** conceptualization (equal), methodology (equal), project administration (equal), software (equal), supervision (equal), writing – review and editing (equal). **Regis Thedin:** data curation (equal), investigation (equal), software (equal). **Michael Lanzone:** resources (equal). **Melissa A. Braham:** data curation (equal). **Tricia A. Miller:** conceptualization (equal), data curation (equal), methodology (equal), writing – review and editing (equal). **Christopher J. Farmer:** conceptualization (equal), funding acquisition (equal), methodology (equal), writing – review and editing (equal). **David Brandes:** conceptualization (equal), methodology (equal), writing – review and editing (equal). **Todd Katzner:** conceptualization (equal), funding acquisition (equal), methodology (equal), writing – review and editing (equal).

## Funding

This work was supported by U.S. Department of Energy Office of Critical Minerals and Energy Innovation Wind Energy Technologies Office.

## Conflicts of Interest

T.M. has a personal relationship with the owner of the company that produces the animal tracking devices used in this study. C.F. is employed by a company that develops and operates wind facilities in the United States and globally.

## Figures and Tables

**FIGURE A1:**
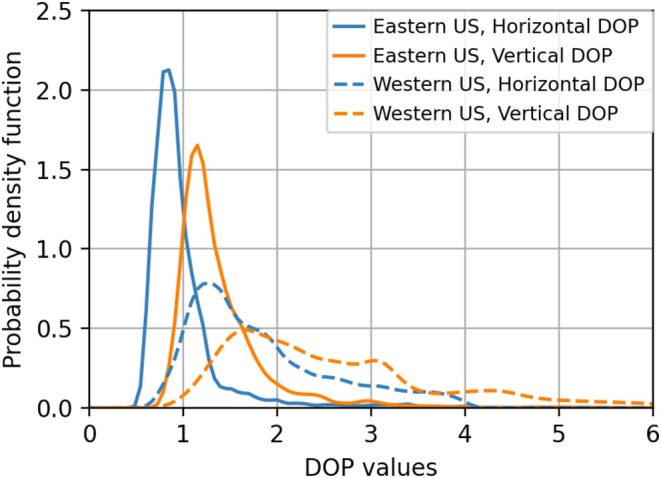
Probability density function of DOP values provided by the telemetry unit for the horizontal and vertical fix, considering only the track data from the western and eastern United States.

**FIGURE A2:**
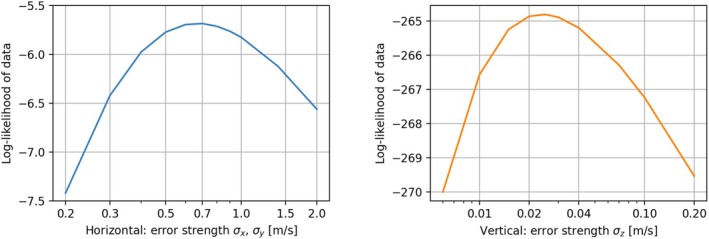
Maximization of Log‐likelihood to obtain optimal process error strengths $\sigma_{x}=\sigma_{y}$ and $\sigma_{z}$.

**FIGURE A3:**
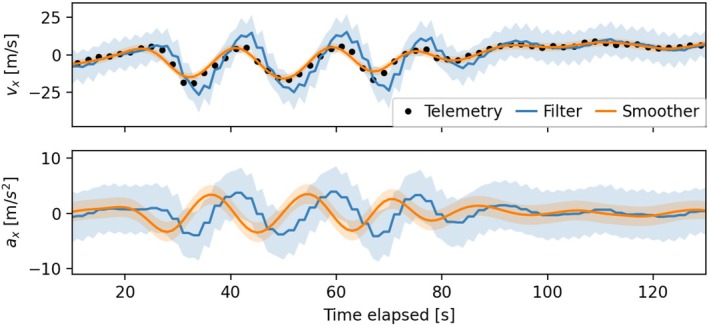
Kalman filter and Kalman smoother estimates of velocity $v_{x}$ in x direction for a sample 2‐min track segment. The confidence bounds pertain to ±3 of the standard deviation of the updated state distribution of $v_{x}$.

## Data Availability

The telemetry data were provided under a non‐disclosure agreement with Conservation Science Global and is not publicly available. The code used to produce the results reported in this paper is provided at github.com/rimplesandhu/raptor‐lookahead‐movement‐model.

## Appendix A Kalman Smoother

Kalman filtering uses iterative Bayesian updates to assimilate noisy positional observations from telemetry data with a stochastic state‐space motion model. This assimilation results in a smooth time history of movement dynamics (position, velocity, heading, etc.) and the associated uncertainties (Särkkä 2013; Grewal and Andrews 1993) in the form of a time‐evolving multivariate Gaussian distribution. Kalman filter considers only the present and past data for estimating the probability distribution of the state, while the smoother uses all the data (past, present, and future) at each time step. We will later discuss how this difference between the filter and smoother affects the state estimation of movement dynamics.

We chose the stochastic constant acceleration (SCA) model as the continuous‐time motion model for each direction in the 3D Cartesian coordinate system. The model assumed a constant acceleration and the temporal change in acceleration is modeled as a white‐noise process. Or, mathematically speaking, ∂a/∂t=w, where a is the acceleration, t is the time, and w is the zero‐mean white‐noise process with strength σ. Subsequently, velocity v and position p were modeled as ∂v/∂t=a and ∂p/∂t=v, respectively.

Discretizing the continuous‐time SCA motion model using the Euler‐Muryama scheme resulted in a state‐space form (Kloeden and Platen 2011)

(A1)
$$\left\{\begin{array}{c} p_{k}\\ v_{k}\\ a_{k} \end{array}\right\}=\left[\begin{array}{ccc} 1 & T & T^{2}/2\\ 0 & 1 & T\\ 0 & 0 & 1 \end{array}\right]\left\{\begin{array}{c} p_{k-1}\\ v_{k-1}\\ a_{k-1} \end{array}\right\}+q_{k}\Rightarrow x_{k}={\mathrm{Ax}}_{k-1}+q_{k},$$

where T∈ℝ is the time‐invariant time interval, $x_{k}\in {\mathbb{R}}^{3\times 1}$ is the state vector at time $t_{k}\in \mathbb{R}$, $A\in {\mathbb{R}}^{3\times 3}$ is the process dynamics matrix, and $q_{k}\in {\mathbb{R}}^{3\times 1}$ is the process error vector with zero mean and covariance matrix $Q\in {\mathbb{R}}^{3\times 3}$ computed as

$$Q_{k}=\sigma^{2}\left[\begin{array}{ccc} T^{5}/20 & T^{4}/8 & T^{3}/6\\ & T^{3}/3 & T^{2}/2\\ \mathrm{sym} & & T \end{array}\right].$$

This covariance matrix captured the relationship among uncertainties in position, velocity, and acceleration, at a time resolution of T seconds. Note that σ has the units of speed, that is, meters per second.

The observation equation related the observed variable (i.e., position) to the state vector x and was formalized in a state‐space form as

(A2)
$$y_{k}=\left[1\;0\;0\right]\left\{\begin{array}{c} p_{k}\\ v_{k}\\ a_{k} \end{array}\right\}+r_{k}\Rightarrow y_{k}={\mathrm{Hx}}_{k}+r_{k},$$

where $y_{k}\in \mathbb{R}$ is the position observation from the telemetry data, $H\in {\mathbb{R}}^{1\times 3}$ is the observation‐state matrix, and $r_{k}\in \mathbb{R}$ is the observation error. We assumed $r_{k}$ to be additive, zero‐mean, and distributed as a Gaussian with strength γ.

Using the motion model in Equation (A1) and the observation model in Equation (A2) we executed independent instances of Kalman smoother for x (easting), y (northing) and z (altitude) directions. Table 1 lists the values for model parameters. The error strength γ of position observations in each degree of freedom was computed by multiplying the time‐varying dilution of precision (DOP) value provided by the telemetry unit, describing the quality of GPS fix, with a time‐invariant DOP multiplier (fixed for a given telemetry unit). Figure A1 shows the distribution of DOP values (horizontal and vertical) for the eastern and western US data, considering only the track data. Higher DOP values for the eastern data were due to the use of older telemetry units. Based on the DOP multipliers in Table A1 and the DOP values in Figure A1, we observed errors of about 2 m in the horizontal position and about 6 m in the vertical position.

**TABLE A1:** Parameter settings for Kalman Smoothern.

	x, Northing [m]	**y**, Easting [m]	**z**, Altitude [m]
DOP multiplier	2.5/$\sqrt{2}$	2.5/$\sqrt{2}$	4.5
Process error strength, σ	0.7	0.7	0.025
Start state covariance matrix, Q	Diag ([5,5,2])	Diag ([5,5,2])	Diag ([5,5,2])

*Note:* Observation error strength, σ = DOP (from telemetry unit) × DOP multiplier. Diag(s) represents a diagonal matrix with column s.

The process error strength σ is required for each direction to compute $Q_{k}$ in Eq. Appendix A. A higher σ value will produce a relatively flexible SCA model that will attempt to consider the observation noise as valid movements. On the contrary, a lower σ will produce a rigid SCA model that will fail to capture sudden valid maneuvers. We obtained an optimal value of σ by maximizing the likelihood of data, which quantifies the probability of observing the data given the model and its parameters. We first computed the logarithm of the likelihood of data for each data point, then take its mean within a track, and then take the mean across all the tracks. We refer to this variable as the *log‐likelihood of data*. The mean log‐likelihood value for x and y directions was further averaged since DOP values for x and y are the same (see Table 1). In other words, we assumed $\sigma_{x}=\sigma_{y}$. Figure A2 shows the variation in log‐likelihood values for varying process error strength values. The optimal values obtained by maximizing the log‐likelihood are shown in Table 1. A lower value of $\sigma_{z}$ in comparison to $\sigma_{x}$ or $\sigma_{y}$ was because the horizontal motion had a more temporal variation than vertical movements for golden eagles, or, raptors in general.

Next, we explored the difference between the Kalman filter and Kalman smoother. Figure A3 shows the filter and smoother estimates of velocity $v_{x}$ and acceleration $a_{x}$ in x (easting) direction for a sample 2‐min stretch of a track. The telemetry data for $v_{x}$ shown in Figure A3 was computed using the instantaneous heading and horizontal speed provided by the telemetry unit. The telemetry data for $v_{x}$ was at variable time intervals whereas the filter/smoother results were at a constant 1‐s resolution. Only the position observations in the x direction were used to execute Kalman filtering.

The temporal lag between the filter and smoother estimates of $v_{x}$ was because unlike smoother the filter is not aware of future movements. Therefore, the filter had a delayed response in its state estimates following sudden movements. This temporal lag was much more pronounced for the acceleration $a_{x}$. Also, the uncertainty in smoother estimates was lower because it uses more data than the filter. These differences between the filter and smoother demonstrated that the smoother was a better alternative when resampling movement data into a constant time resolution while deriving higher‐order movement dynamics.

We found that both cubic spline and Kalman smoother performed comparatively when contrasting the mean resampled horizontal path of a track. Figure A4a shows the Kalman smoother output for a 2‐min segment of a sample telemetry track. We found that a significant difference in performance exists for these two techniques when computing higher‐order variables such as $v_{h}$ and $a_{h}$ (which is used to compute heading rate). We found that the cubic spline estimate of $v_{h}$ mostly tried to follow the telemetry data, while the corresponding Kalman smoother estimate tends to capture the general trend while respecting the telemetry errors (Figure A4b). The performance of cubic spline further degraded when estimating $a_{h}$.
